# Evolution of a research team: the patient partner perspective

**DOI:** 10.1186/s40900-022-00377-3

**Published:** 2022-08-24

**Authors:** Suzanne Madison, Alex D. Colon-Moya, Wilfredo Morales-Cosme, Margie Lorenzi, Aracelis Diaz, Bridget Hickson, Kathy Monteiro, Alexander Muniz Ruiz, Addie Perez, Richard Redondo, Dennis Reid, Janet Robles, Marsha Santiago, Opal Thompson, Joyce Wade, Mary White, Graciela Castillo, Cristian Valenzuela

**Affiliations:** 1 Patient Partner/Advisor, 6337 Alderwood Circle, #B, St. Paul, MN 55129 USA; 2 Patient Partner/Advisor, South Jordan, UT USA; 3 Patient Partner/Advisor, Bayamon, Puerto Rico; 4 Patient Partner/Advisor, Hyde Park, MA USA; 5 Patient Partner/Advisor, Corozal, Puerto Rico; 6 Patient Partner/Advisor, Dorchester, MA USA; 7 Patient Partner/Advisor, Orlando, FL USA; 8 Patient Partner/Advisor, Yuma, AZ USA; 9 Patient Partner/Advisor, Winter Park, FL USA; 10 Patient Partner/Advisor, New York, NY USA; 11 Patient Partner/Advisor, Bronx, NY USA; 12 Patient Partner/Advisor, Boston, MA USA; 13 Patient Partner/Advisor, Euclid, OH USA; 14 American Institutes for Research, 1400 Crystal City Drive, 10th Floor, Arlington, VA 22202 USA

**Keywords:** Patient partner, Patient advisor, Patient engagement

## Abstract

**Introduction:**

Despite a movement toward the inclusion of patient partners or advisors as part of the research team in all funded studies, few publications have discussed patient engagement from the patient partners’ perspective.

**Methods:**

Qualitative interviews were conducted by independent qualitative researchers to collect and summarize the experiences and perspectives of the 16 Patient Partners (PPs) on the study team for PeRson EmPowered Asthma RElief (PREPARE), a large, pragmatic study of 1200 African American/Black (AA/B) and Hispanic/Latinx (H/L) adults with asthma. This study was funded by the Patient-Centered Outcomes Research Institute.

**Results:**

This paper, authored by the PPs themselves, summarizes qualitative interview findings. The journey of the PREPARE PPs began with a desire to learn more about asthma and advocate for other individuals with asthma. Many challenges, including intimidation and lack of trust, were overcome as the research team prioritized building a comfortable environment in which PPs’ lived experiences, opinions, and cultural beliefs are valued, and in which PP voices are centered and respected. Over time, the PPs gained confidence in expressing ideas and feedback, and in taking ownership of their role as valued members of the research team. The PP experience has had tremendous personal and professional impact on the PPs themselves, while also modeling a change in the way researchers and PPs relate to and partner with each other.

**Conclusion:**

The partnership between PPs and researchers in the PREPARE study has elevated the PP role from external advisors to integral and empowered members of a collective research team, and the partnership developed and evolved over time.

**Supplementary Information:**

The online version contains supplementary material available at 10.1186/s40900-022-00377-3.

## Plain English Summary

Patients and caregivers who have lived with a condition have an important and unique perspective researchers should consider. To this end, patients and caregivers have expanded their involvement in the design and conduct of clinical research, joining research teams and being engaged in the research process from start to finish. Researchers have reported on the positive impact that these “patient partners” or “patient advisors” have on research. But few papers report on the impact on the patient partners/advisors from being on a research team.

Here we report the lived experiences of the 16 Patient Partners who served on the research team for a large study of 1200 African American/Black (AA/B) and Hispanic/Latinx (H/L) adults with asthma. These Patient Partners offered their input over 7 years, spanning from study design, study conduct, understanding results, to sharing results.

To understand the Patient Partners’ experiences, we conducted interviews asking about their journey on the research team.

- Motivations and reasons they became Patient Partners
- Initial experiences with the research team
- If and how over time, they became comfortable and developed confidence
- If and how the research team made them feel valued and respected
- The personal and professional impact of being a Patient Partner
- Their advice to researchers and fellow patients considering including or joining as Patient Partners or advisors

By reporting the perspectives of these 16 Patient Partners, we hope to support continued movement toward broader and better inclusion of patients and caregivers on research teams.

**Supplementary Information:**

The online version contains supplementary material available at 10.1186/s40900-022-00377-3.

## Introduction

Patients’ and caregivers’ lived experience with a condition or illness gives them unique expertise and perspective, and their participation and cooperation are required for successful completion of clinical studies and trials. It thus seems both prudent and appropriate that clinical research would include patient advisors or partners as part of the research team, helping to center and focus the research and its design on what is of greatest importance to patients and their caregivers.

Over the past decade, several funding agencies have developed guidelines and requirements for the inclusion of patient partners as part of the research team in all funded studies [1–5] and many publications have addressed why this is important and potentially valuable [6–9]. Other publications discuss how to engage patient partners [4, 10–14]. with some including metrics to assess how and how often patients are engaged [13, 15–21]. Most publications focus on the impact of the patients on the study design and conduct or the work and perceptions of research team members [22–27]. Few works have been published discussing patient engagement from the patient partners’ perspective. Specifically, few have addressed why individuals choose to be engaged, how their level of engagement and comfort in being part of the team may have evolved over the period of the study, and how working on the study team may have impacted them personally or professionally [28].

This report addresses those gaps in the literature by talking with and presenting from their perspective the experiences of individuals who participated as “Patient Partners” (PPs), also sometimes called patient advisors, on the study team for PeRson EmPowered Asthma RElief (PREPARE) [29], a large, pragmatic study of 1200 African American/Black (AA/B) and Hispanic/Latinx (H/L) adults with asthma. By better understanding the perspectives of PPs from various cultural and ethnic backgrounds, we can support the movement toward more and better engagement of these groups of patients on clinical research teams. In this manuscript, written by the PREPARE PPs, we present findings from independently conducted qualitative interviews in order to share our experiences and perspectives as part of the PREPARE study team.

## Materials and methods

### Overview of the PREPARE study and the patient partner role

The PREPARE Study is a randomized, open-label, pragmatic study which aims to assess whether a Patient-Activated, Reliever-Triggered Inhaled CorticoSteroid (PARTICS) strategy can improve asthma outcomes in AA/B and H/L adults with moderate to severe asthma. Details of the PREPARE study design and methods have been published previously [29].

The PREPARE research team sought to implement their goals plus PCORI recommendations and requirements to ensure that PREPARE remain patient-centered by involving PPs at all stages of research, including study design, grant submission, implementation, data analysis and interpretation and dissemination of the results. The approach included having PPs help identify and investigate outcomes that matter to patients; develop an intervention designed to be simple, practical, and patient-empowering; and develop and adopt study methodologies that are of low burden to study participants. To achieve the goal of patient-centeredness, the research team relied heavily on guidance from the PPs, who are all AA/B or H/L adults with asthma and/or caregivers of individuals with asthma. Along with other PREPARE stakeholders (e.g., healthcare professionals, representatives of professional societies, health policy leaders, and clinical trials experts), the PPs are an integral part of the research team and have been involved in all aspects of not only planning but also decision-making throughout the study.

### Patient partner recruitment and demographics

The initial group of PPs was identified and recruited by the academic investigators. Knowing the need for a broad-based group of PPs to provide input, the investigators and other members of the research team reached out to English- and/or Spanish-speaking AA/B and/or H/L individuals, who they knew personally as adults with asthma, caregivers of children or adults with asthma, or community members and activists interested in improving asthma management on the local level. Initially, these individuals were invited to attend a focus group to learn more about the PREPARE study and determine their interest in becoming PPs. Recognizing gaps in distribution in the selected PPs’ geographic location and preferred language (English vs Spanish), the group was expanded through referral from the PPs themselves, PREPARE clinical site investigators and other members of the stakeholders group.

Currently, the PPs include 16 individuals (11 female, 5 male) representing 8 states primarily in the Eastern half of the US and the US territory of Puerto Rico (in line with the geographic distribution of the PREPARE sites). Six PPs self-identify as AA/B, 9 self-identify as Hispanic/Latinx, and 1 self-identifies as Native American and AA/B. Ten PPs speak and prefer English while the other 6 speak and prefer Spanish. Twelve PPs have asthma and 9 are caregivers of someone with asthma, which includes 5 who serve both roles. Ten had participated as a patient advisor or stakeholder in a prior study.

### Patient partner involvement in PREPARE

PPs have been part of virtual meetings held at least monthly throughout protocol development, participant enrollment, and follow-up. They will continue meeting through data analysis, interpretation, and results dissemination. For these meetings, PPs are divided into two groups by preferred language spoken: English (n = 10) and Spanish (n = 6). Representatives from the AA/B and the H/L PP groups are included in monthly meetings of the PREPARE Executive Committee and other committees, such as the Questionnaire, Publications and Ancillary Study Committees. All PPs are included in the in-person PREPARE annual meeting (meetings were held virtually in 2020 and 2021 due to COVID-19). When required, such as for the PREPARE Annual Meeting or other committee meetings, translation is provided for Spanish-speaking PPs. PPs are compensated at the same hourly rate as members of other stakeholder groups.

To ensure that PP voices are heard, and their input is brought back to the Operations team, PP engagement staff are present at all meetings. The PP engagement staff includes a physician researcher with experience in patient engagement in pragmatic trials, a PhD nurse researcher with expertise in patient engagement, and a research nurse with extensive experience in engaging patients from minority backgrounds in clinical research. Their work includes maintaining regular contact with the PPs to foster continuing engagement, assuring that most if not all PPs provide input into all aspects of research design and implementation, and problem solving on all calls and email interactions. In addition, the engagement staff ask for regular feedback and provide information and education to the PPs related to research structure and function such as inclusion and exclusion criteria, blinding, randomization, ways to review and analyze data, and interpretation and dissemination goals and gaps.

### Data collection and analysis

In order to understand, summarize, and report the lived experiences of the PREPARE PPs, qualitative interviews were conducted by AIR (American Institutes for Research), a non-profit evaluation organization which specializes in qualitative interview techniques. Questions for the qualitative interviews were developed by a team of PPs and the protocol was reviewed and approved by the Mass General Brigham Institutional Review Board (IRB). After obtaining verbal informed consent, two qualitative researchers conducted nine verbal interviews with a total of 15 participants, and one PP submitted written answers to the interview questions. Interviews were conducted in October 2020. Due to project timeline and budget constraints, participants were grouped together for interviews. The majority of the interview sessions were with two interview participants, one included three participants, and a few were one-on-one interviews. Several steps were taken to ensure that participants were comfortable speaking freely in groups. First, the PREPARE study team coordinator, who worked closely with the group and knew the participants well, thoughtfully grouped participants based on the rapport and compatibility they had with each other. Second, prior to beginning the interview, interviewers asked participants if they were comfortable in a group setting and aimed to establish parameters for a safe discussion. Interviewers let participants know there were no correct or incorrect answers, that the team was very interested in their opinions and experiences, and that anything discussed in the interview should not be shared outside of the interview. Throughout the discussion, interviewers used neutral probes to encourage a response from both participants. Interviewers observed that all participants were comfortable talking with each other and sharing experiences and did not seem to strongly influence or constrain each other during the interviews.

Interviewers used a semi-structured interview guide addressing three general topic areas: the PPs’ journey during PREPARE, the personal and professional impact of being a PP, and the PPs’ advice to researchers and fellow patients regarding conduct and inclusion of PPs in research. Following interview completion, AIR researchers sorted the transcribed and written interview responses into these three general topic areas. Applying the principles of deductive thematic analysis, each AIR researcher individually grouped responses per topic area by similarity or difference to identify themes and patterns. Agreement between staff was reviewed and verified. Representative quotations were then selected to illustrate themes and verified by agreement between AIR researchers. Patient and public involvement in this study, using the GRIPP2 Short Form, is published along with this article as an Additional file 1.

Below are the collective words of the PPs themselves summarized through the interview findings.

## Results

PP advice and feedback has informed nearly every aspect of the PREPARE study, from selecting the primary outcome measure and the magnitude of change considered clinically significant, to providing plain language and cultural review of the questionnaires, phone scripts, video scripts, and graphics used in the study. PPs have further helped to troubleshoot issues as they arise during the study, such as the low questionnaire completion rate observed in the pilot study [30].

### The patient partners’ journey during PREPARE, in their voice

#### Motivations and reasons to become a patient partner

There are many reasons that we were motivated to accept the invitation to become a PP, as outlined in Table 1. Many of us wanted to learn more about treating and living with asthma, because we either have asthma or care for someone who does. For some of us, gaining experience in research is relevant to our current job or educational experiences. Financial compensation, while not the primary reason that we chose to participate, is appreciated and makes a positive difference in many of our lives. Perhaps most importantly, we saw the opportunity to help researchers understand what it is like to live with asthma, and “*ponerle voz a la gente que en ocasiones no tiene voz* [to give a voice to people that sometimes do not have a voice].” We hoped this research would help many people—including the PREPARE study participants as well as individuals with asthma beyond this study—and that it would impact future asthma research and researchers.

**Table 1:** Patient partner interview quotes: the patient partner journey

**Motivations and reasons to become a patient partner**
*For me, the asthma condition and helping people how to live with asthma*. –Respondent 12
*When I had an interview with a* [Patient] *Partner, I saw the opportunity to give a voice to people who sometimes have no voice. Because precisely the study is focused on Latinos, who are a minority, and Afro-Americans, who are also a minority.* –Respondent 16
*And we hope that hopefully, once the research is over, we have helped that many more people, and we have collected a whole lot of data too that helps out with other researchers*. –Respondent 7
*But, the realistic fact, if I wasn't being paid, because I do a lot of volunteering in my community, I probably would've participated still because I wanted to know more about the steps, and when you're doing a study in how my voice could make a difference and my input. I just wanted to make a difference. So, I would've probably still been involved. But realistic, as a mom with children, having that funding, that is something to look forward to, because that's a bill for me. That can contribute to food, or other issues, medical costs, household things that I needed. So that funding was really, it was a plus*. —Respondent 2
**Initial experiences with the research team**
*And in the first meeting, too, we do like the small breakout groups. And I remember I was put in one with* [the PREPARE Principal Investigator] *and he … I was kind of shy to answer, and he said, ‘You as a patient partner*, [Respondent 1], *what do you think?’ And so, I was like, ‘You want to know what I think? Okay.’ So, it was nice that even though I was kind of shy and quiet he sought me out and was like, ‘No, we want to hear from you. What do you think?’ Even, in breakout groups, I felt like my input was wanted and needed, welcomed in a comfortable environment*. –Respondent 1
*Those who did not understand English well and preferred an interpreter*, [the research team] *accommodated them with their headphones and a professional interpreter*. –Respondent 15
**Getting comfortable, feeling valued and respected, and evolving to confidence in the PP role**
*You could call* [members of the PP engagement staff] *or any of the other members. Everyone was accessible*.
*Everyone had a voice…everyone’s participation meant something*. –Respondent 3
[Being invited to meetings and given all the details] *makes me feel like they really want me there, they want to hear what I have to say*. –*Respondent 6*
*I know these are just regular people, these are regular people in suits. But it took a couple times meeting with them in board meetings and seeing their presentations and seeing, or even how they talk about their site workers. Like just the way they talk about how much they value them, and how recruitment is so good because we have this Spanish speaker we hired, five Spanish speaking people to enroll patients, and it's just been so great since then because they have culture piece. So just knowing that they valued that cultural piece. They value that, they didn't straight up say, ‘Hey, I really value this cultural piece.’ But just the way that they talked about it*. –Respondent 1
*You get a sense of value…that you’re valued because simply by paying an airline ticket*. –*Respondent 8*
*I feel more comfortable now because we have more confidence. We talk more, I know them, and they are like my family. I feel good*. –Respondent 12
*What surprised me over time is just how, I just thought we were just kind of like pieces to a puzzle, but I didn't realize how big of a piece we were*. […] *People are interested in what we have to say, and they are interested in how we're saying is going to affect the study. So that made me feel like, wow, professionally I don't feel this way all the time, but they go out of their way to make sure that we feel like we are valued participants and have something important that they want to know from us*. –Respondent 1
…*second family, an asthma family. Our research asthma family*. –Respondent 4

#### Initial experiences with the research team

At the beginning, there were challenges. Many of us did not have any experience working directly with a research team, and there were different levels of understanding among us. We felt insecure around the researchers and physicians, especially when faced with terms and jargon that we didn’t understand. We did not want to seem ignorant or uneducated and so did not always ask for clarification. At the beginning, we were unsure what our roles and responsibilities were, and how we were going to be able to contribute to the study in a meaningful way. We also had some lack of trust in the research process and were concerned about the possibility of the study having a negative impact on the study participants, or even us.

The first time we met in person with the researchers at the initial PREPARE annual meeting, even after having interacted on phone calls for a number of months, we experienced a mixture of feelings, including intimidation, confusion, nervousness, and also happiness, excitement, and a sense of purpose. A few of us had never traveled alone before, and the process of traveling by plane to the East Coast caused nervousness. To support us, a member of the research team was available to provide guidance with regards to travel, and “*they don’t make you feel uncomfortable if you don’t understand.*”

#### Getting comfortable

Once we arrived at the meeting, there were several ways in which the researchers helped to build a comfortable environment in which we could interact freely (Table 1). First, we were asked to share our photograph and personal details, which helped us feel welcomed and centered. Second, the research team made us feel that our voices were important and respected by directly asking for our thoughts and being clear that there was no wrong answer to any question. *“I don't feel like they're more superior, and they don't make me feel that way. So yeah, things have changed a great amount. I think once you get to know, we're all there because we want to know about asthma, so the realistic fact is we all have something in common. That's how I look at it.”* In addition, we have been able to ask for what we need, and those requests have been granted (for example, PP-only or PP-focused meetings in which we could ask questions we might not feel comfortable asking in the larger group; technical support for internet connection issues; WhatsApp reminders for virtual meetings). Finally, for those of us who speak Spanish, having all materials translated and a professional English/Spanish translator has been very important to us feeling included and valued, knowing that language would not be a barrier.

Having monthly calls and in-person meetings has supported building a strong relationship among us as PPs, and with the other research team members. During calls, the host constantly emphasizes how important our opinions are for the researchers, and research team members are responsive and active listeners. In addition, the Principal Investigator and other co-investigators join the calls from time to time to give us updates on the project, discuss topics related to asthma, and address our concerns. Finally, we continue to meet in person every year at the PREPARE annual meeting (with the exception of virtual meetings in 2020 and 2021 due to COVID-19), and we notice that each year we grow more confident, and our bonding becomes stronger, to where we now feel like part of a “*research asthma family*.” Overall, the accessibility of the researchers has helped relieve our anxieties and concerns and has allowed for the creation of a comfortable environment.

#### Feeling valued and respected

Respect has been a crucial and binding element in the relationship between PPs and the other members of the research team and has been central to us feeling valued as colleagues (Table 1). We are treated as professionals and compensated accordingly, included in committees (such as the Executive Committee), invited to participate in presentations and meetings, and given all the study details. In every interaction, the researchers take seriously all our comments and feedback, and later they report back to us on how our comments were used. *“Obviously there are people who are experts in their area, but the best thing about meetings is that everyone's opinion is important to them. The academic specialty is not important, everything is told, everything is listened to, everything is analyzed, and everything is respected."*

#### Evolving to confidence in the PP role

Now, after several years as PPs, we feel invested in the study, and are excited to learn about and help interpret the findings and to be part of disseminating the results. We have improved our own knowledge about asthma, but more importantly, we know that our input has brought value to the PREPARE study and its participants and can help improve other people’s experience with asthma in the future. We feel valued and feel that we are a part of something bigger which will have impact beyond our immediate circles. *“I felt worthy. It made me feel really important. I was glad to be a part of something so big, and I was glad that my voice was heard, and it made people listen. So, I really enjoyed that.”*

### Personal and professional impact of being a PP

The journey and experiences of being a PP have impacted not just the study and the research team but also us, both personally and professionally (Table 2). On the level of personal health, many of us have learned about asthma and seen our own asthma health improve as we gained a better understanding of asthma triggers and how to use asthma medications correctly. On the level of public health, we have grown in our knowledge of the ways in which certain populations are impacted differently or have less access to medications or healthcare. Armed with better understanding, we feel even more empowered to advocate for those in our communities whose voices may not be heard because of the inequities they experience.

**Table 2:** Patient partner interview quotes: personal and professional impact of being a patient partner

**Personal impact of being a patient partner**
*I turned myself into an asthma leader, which I am. A parent asthma leader, and I wanted to just get the word out there. And I worked at my daughters’ schools, and I just did everything possible. *[…]* I want to see the emergency room visits go down. So, everything about this was me, for me. I enjoyed it. I wanted to do it. It was something that meant something to me. And I want to make a difference in the world and in our community with sick people with asthma.* –Respondent 4
*I really appreciated that everybody’s so diverse, in their experience and in their talents. And I think maybe being in the study has brought out talents that they didn’t even know they had*. –Respondent 8
*Being involved* [in the study], *I'm asking more questions and feeling more confident. So, I've been really grateful to be part of this study because I've actually got a lot of, a lot of knowledge in the right stuff*. –Respondent 2
**Professional impact of being a patient partner**
*It has opened up so many opportunities. As a Patient Partner, you get to meet all the researchers, all the doctors. Everybody plays a role in that research. And now we're expanding* […], *they’re bringing us out into other research, and to the possibility of other people that we can help, besides asthma*. –Respondent 7
*Professionally, it has helped me to have more confidence in myself. In knowing how to communicate with different people who have different titles. And, as* [Respondent 16] *said, we all have our specialty, and we all have a grain of sand to contribute. The important thing about this group is that I have never felt that, because such a person is not a doctor, that opinion* [was] *not taken into consideration*. –Respondent 15
*But as a professional, you take away a lot. You're at a different level, definitely. It's like, how can I say it, like a celebrity. Like a celebrity, right? Because at the end, once they complete the research and everything, we have credits. They'll give all of us credit for participating in that research. That means everybody that was part of that research will have some type of acknowledgement. So, we all played, even if it was a small part into that study, we all did that together, as a team*. –Respondent 7

We have all been impacted professionally in some way, whether it be skill development, networking, or exposure to new opportunities. Because the PREPARE project team includes experts from many different fields, we have been able to critically challenge and engage with one another. For those of us whose professional work intersects with the community and their struggles, needs and strengths, our firsthand experience as PPs in this study—including the impact of personal, honest, and reliable communication and feedback—has informed the way we engage with the people we serve. For others, particularly those working in healthcare or research, the experiences and knowledge gained as PPs have enhanced our work. And in general, most of us have gained confidence in being able to interact with a variety of professionals and comfortably express our opinions.

Finally, the PP experience has been incredibly personally rewarding. We have had the unique opportunity not only to share our own personal lived experiences, but also to share the knowledge and experiences of those we know or have worked with who do not have a platform to share themselves. We have been able to take part in spreading the awareness and consciousness necessary to bring about change within the medicine and health industries, including having necessary, crucial conversations surrounding oppression, race, language, and other ethical and cultural concerns. Considering all of this, we are proud of our participation and contributions to a great cause by sharing our life experiences, knowledge, wisdom, advice, and concerns. We have gained confidence in knowing that our opinions are valuable and that through sharing our voices, change is certainly possible not only on the local level, but also on the broader and more complex institutional levels of the practice of medicine.

### Advice to researchers and those considering becoming patient partners or advisors

From our experiences as PPs in PREPARE, we offer suggestions to both researchers and future PPs (Table 3) that we believe will help in future research projects.

**Table 3:** Patient partner interview quotes: advice to researchers and those considering becoming patient partners or advisors

**Advice to researchers**
*I think that they need to have Patient Partners because it depends on what community you're dealing with, because when you're dealing with the Black community, they do things a little different, when you're dealing with the Latinos, they're pretty much close to the Black, but when you're dealing with the like of Vietnamese and different cultures that don't do things the way we do things, they need to have a person from that community a part of the research. So, everybody doesn't do things the same in each community, in each race, or they do things a little different*. […] *So just being versatile with the people that you are researching, just try to have a person that's a part of the same community and the research*. –Respondent 15
*Be like* [the PREPARE Principal Investigator]. *I feel like he is very open to listening to our stories, of “this is how it is for us,” and what he's seeing with his patients. So, I feel like he's very good* […] *and don't doubt of the competence that the patient partners have*. –Respondent 2
**Advice to those considering becoming patient partners or advisors**
*I would say, if you have the opportunity, to engage. I believe that we are here on this earth to give back into the lives of others, especially people that are suffering or going through battles medically. So, I would say, ‘patients, hey, if you get the opportunity to get involved with a clinical trial, asthma or whatever, do it.’ Because, in that way, the patient is literally reaching into someone else's life and with the goal to help. To give them all the knowledge and the wisdom and the peace of mind, and so forth, as they're going through. You have to give back*. –Respondent 10
*Go in with an open mind and be willing to learn and listen. That's about the best advice I can do, because if you go in with an open mind, you're going to hear stuff, you're going to learn more and you're more open for it. Just be open to listen. Listen to other people*. –Respondent 15
*Journey is amazing, it will change your life. Just being a participant, being part of it, it will make a difference. Don't be afraid, ask questions. There's no stupid question*. –Respondent 2
*And I think that's the key to everything, is knowing the people in your group, taking that time to be personable, care about people. Unfortunately, in this world, there's not enough of that going on in everything, just ask somebody how they're doing, little stuff like that is very important. But I think, for us, we have a really good group and I pray that everybody else would have the same experience that we have, but I really like looking, care about and pray for everybody in my group and their wellbeing and stuff. It really is a second family to me*. –Respondent 5

#### Advice to researchers

Researchers, it is vital to include PPs in studies, especially when working with communities of color or other underrepresented populations. Our lived experiences as AA/B and H/L people in a white dominant society means that you will need to invest extra effort to engage us in your studies. We expect you as the research team leaders to proactively and aggressively support us in being fully informed regarding all aspects of the study. You might consider offering educational opportunities to help us become familiar with things important in research design, such as ethical research conduct, informed consent, patient privacy, and study regulations and procedures. Avoid using terms or other jargon that we may not understand. The return on your investment will be less fear and mistrust and potentially improved participant enrollment and study completion. This is especially important among people of color and other marginalized groups.

To keep us engaged in your studies, we must feel comfortable, included, heard, and valued regardless of our age, race or ethnicity, profession, or level of expertise or knowledge. Equally important is that our value does not diminish over time. We suggest that researchers promote a peer-to-peer relationship with your patient advisors, in which there is a sense of equality in decision-making power, access to resources, and input into the study design, implementation, results interpretation and dissemination of results. Keep us informed about meeting schedules and shared responsibilities. In addition, while financial compensation is usually not the primary motivation for recruitment and retention in clinical trials, but just as all investigators are paid for their work and expertise, we expect appropriate compensation for our time and expertise.

#### Advice to those considering becoming patient partners or advisors

Fellow patients, when we were asked to be Patient Partners in a research study of adults with asthma, we were hesitant. As men and women of color, especially during these trying times of political and civil unrest, the COVID-19 pandemic, and now vaccine distribution, any mention of participating in research might easily bring to mind what we know of the past abuse of communities of color by some members of the research community. We understand; we were you. But as we became truly engaged and supported as members of the research team, we developed a trust in the process, and in the researchers. You can also develop trust as you realize that you are helping other patients, their caregivers, and their families by helping researchers ensure that the results are meaningful to people like us.

It is important to understand that when you participate as a PP, your job is not easy. Your PP experience may be enhanced and more comfortable if you are able to educate yourself on what it means to be part of a study team, perhaps through online resources [31]. Your role has responsibilities that include a commitment of time and effort—perhaps over several years. Keep in contact with the research team if you move or change your phone or email address. Show up for meetings or ask about rescheduling when life gets in the way.

We suggest advocating for opportunities to engage with your fellow PPs. Being a PP can be a stressful process and sharing your common experiences will provide support and a sense of community. Having a strong connection to one another will help you feel more comfortable engaging with the research team, allow you to share with more honesty and vulnerability, and help you cope with stressful situations.

Let go of preconceived notions; be prepared to be open to listening and learning, and be prepared to actively engage. And know that the reward will be worth the effort. *“*[The] *journey is amazing, it will change your life. Just being a participant, being part of it, it will make a difference. Don’t be afraid, ask questions. There’s no stupid question.”* We hope that you will consider raising your voice as patient advisors, as you will provide a positive impact for yourselves, as well as for your communities and countless others who may suffer with a medical condition.

## Discussion and conclusion

The PREPARE Patient Partners are a group of 16 AA/B and H/L men and women who had the courage and opportunity to embark on a journey with the PREPARE researchers—a journey that has had tremendous personal and professional impact on the PPs themselves, while also modeling a change in the way researchers and PPs relate to and partner with each other. This partnership has elevated the PP role from external advisors to integral and empowered members of a collective research team, and it has developed and evolved over time.

The PP journey began with a personal desire to learn more about asthma and an equally important motivation to help researchers understand what it is like to live with asthma in the AA/B and H/L communities. Trust in the research process and the researchers evolved as PPs developed a sense of being valued for the significance of lived experiences, and respected for opinions, feedback, and cultural beliefs. Through the experience of being a critical member of the PREPARE research team, offering input and guidance at each step of the research process, PPs have taken ownership of their roles, grown more confident in expressing ideas and feedback, and become more knowledgeable about asthma—ultimately resulting in a deeper sense of empowerment in advocating for themselves and others. It is with this knowledge that we are able to provide advice to researchers and other patients on the role of PPs within research studies.

We conclude that the partnership between PPs and researchers is a complex interplay and an opportunity for clinical research to become more inclusive, collaborative, and impactful for patients and their communities.

## Supplementary Information

**Additional file 1**. Patient and public involvement in this study according to the GRIPP2 Short Form.

## Data Availability

The data that support the findings of this study are available from the corresponding author upon reasonable request.

## Abbreviations

AA/B: African American/Black
AIR: American Institutes for Research
H/L: Hispanic/Latinx
IRB: Institutional Review Board
PARTICS: Patient-Activated, Reliever-Triggered Inhaled Corticosteroid
PCORI: Patient-Centered Outcomes Research Institute
PP: Patient Partner
PREPARE: PeRson EmPowered Asthma RElief
US: United States
