# Supplementary material for: Evolution of a research team: the patient partner perspective
Source: Res Involv Engagem. 2022 Aug 24;8:42. doi: 10.1186/s40900-022-00377-3 (PMC9400573; doi:10.1186/s40900-022-00377-3)
Supplement: Supplementary file 1 — Additional file 1. Patient and public involvement in this study according to the GRIPP2 Short Form. [file 40900_2022_377_MOESM1_ESM.docx]

**Patient and Public Involvement in the Study using the GRIPP2 Short Form**

| **Section and topic** | **Item** |
| --- | --- |
| 1: Aim  Report the aim of the study | To understand, summarize, and report the lived experiences of the 16 Patient Partners (PPs) who served on the research team for a large study of 1200 African American/Black and Hispanic/Latinx adults with asthma |
| 2: Methods  Provide a clear description of the methods used for patient and public involvement (PPI) in the study | The PPs proposed the concept and design of this follow-up study regarding PP engagement and impact to the research team. Qualitative interviews of the 16 PPs were conducted by an independent organization (American Institutes for Research [AIR]). Questions for the qualitative interviews were developed by a team of PPs. Two qualitative researchers from AIR conducted the interviews of the 16 PPs. Following interview completion, AIR researchers sorted the transcribed and written interview responses into three general topic areas and selected representative quotations to illustrate themes. Using the summary provided by AIR, a team of PPs collaborated to write this manuscript and make final PP quote selection, and all 16 PPs are included as co-authors. |
| 3: Results  Outcomes—Report the results of PPI in the study, including both positive and negative outcomes | - The PPs were able to share their experience among themselves and determine the concepts they wanted to convey to research teams and other potential PPs through this manuscript. - Developing, writing and reviewing the drafts and final manuscript provided a new experience for most of the PPs. - The PPs learned new skills for interpreting and presenting research results from questions they had proposed. - The PPs learned the breadth of experiences and reflected further on their own experience while summarizing results and preparing this manuscript. |
| 4: Discussion  Outcomes—Comment on the extent to which PPI influenced the study overall. Describe positive and negative effects | The involvement of PPs was critical to this study. The PPs recognized the importance of sharing their perspectives and lived experiences. To that end, they proposed the design and concept of this study and received the support of the broader research team. The PPs heavily influenced the development of the qualitative interview questions, the interpretation of the summary, and the writing of the manuscript, which had the positive impact of ensuring that the interviews and manuscript captured, in a nuanced way, the PPs’ journey on the research team. |
| 5: Reflections  Critical perspective—Comment critically on the study, reflecting on the things that went well and those that did not, so others can learn from this experience | The outcomes of this study show that it is important to allow patients and caregivers who serve on research teams the opportunity to systematically reflect on their experiences, and to capture those learnings so that other research teams can learn from their experiences. Qualitative interviews are an effective modality and should be conducted by individuals who specialize in qualitative interview techniques. While we grouped PPs into groups of 2 or 3 for the interviews due to time and budget constraints, we recommend one-on-one interviews when possible. It was important that the PPs be closely involved in the interpretation of the results and in the development of the manuscript. At this stage, our approach was to involve a smaller “writing group” of those PPs who were interested in participating, along with a few members of the research team who had close relationships with the PPs and could help guide the work. Members of the writing group wrote sections of the manuscript, which was then collated and edited for consistency. This was an effective method that allowed for the manuscript to maintain the “voice” of the PPs. |
